# Individual-specific resting-state networks predict language dominance in drug-resistant epilepsy

**DOI:** 10.1101/2025.11.21.25340716

**Published:** 2025-11-25

**Authors:** Mervyn Lim Jun Rui, Zhang Shaoshi, Shreya Pande, Xue Aihuiping, Ru Kong, Kareem Zaghloul, Sara Inati, Thomas Yeo Boon Thye

**Affiliations:** 1. Computational Brain Imaging Group, Yong Loo Lin School of Medicine, National University of Singapore; 2. National Institute of Neurological Disorders and Stroke, National Institute of Health; 3. Division of Neurosurgery, Department of Surgery, National University Hospital

## Abstract

**Importance:**

Identifying language dominance is a crucial step in epilepsy surgery planning. We applied a precision functional brain mapping approach to estimate individual-specific cortical resting-state networks in drug-resistant epilepsy and predict language dominance.

**Objective:**

To determine whether individual-specific cortical network topography can predict task-based language dominance in drug-resistant epilepsy.

**Design:**

Retrospective case-control study conducted between January 2024 and August 2025.

**Setting:**

Multicentre population-based study including healthy participants from the Human Connectome Project, and participants with drug-resistant epilepsy from the National Institutes of Health (NIH) and the University of Iowa.

**Participants:**

Eligible participants had drug-resistant epilepsy defined by International League Against Epilepsy criteria and were undergoing pre-surgical evaluation. All participants underwent neuroimaging, with a subset receiving concurrent intracranial electrical stimulation during fMRI.

**Main Outcomes and Measures:**

Individual-specific cortical network topography and prediction of task functional magnetic resonance imaging language dominance.

**Results:**

Ninety-one participants with drug-resistant epilepsy were included: 61 (67.0%) temporal lobe epilepsy, 29 (31.9%) extra-temporal lobe epilepsy, and 1 (1.1%) undetermined seizure onset zone. The mean age was 33.0 ± 11.4 years and 50 (54.9%) were male. There were 40 healthy participants with a mean age of 29.0 ± 4.0 years, and 16 (40.0%) were male.

We developed a multi-session hierarchical Bayesian model (MS-HBM) trained on NIH data to estimate individual-specific networks in drug-resistant epilepsy. MS-HBM trained on epilepsy data outperformed group-average networks or MS-HBM trained on healthy participants and generalized well to an independent dataset. During concurrent intracranial electrical stimulation, cortical activation and deactivation aligned more closely to individual-specific networks than group-average networks. Individual-specific language network topography significantly differed across left (mean lateralization index (LI) = 0.165 ± 0.106; area-under-the-curve (AUC) = 0.82), bilateral (LI = 0.056 ± 0.074; AUC = 0.72), and right (LI = 0.023 ± 0.055; AUC = 0.83) language dominance groups (p = 0.002).

**Conclusions and Relevance:**

Our model is publicly available (github link), which may be used to predict language dominance from approximately 10 minutes of resting-state fMRI. This provides a practical, non-invasive tool for presurgical evaluation of drug-resistant epilepsy.

## Introduction

Identifying language dominance is a crucial step in epilepsy surgery planning. Current guidelines recommend that task functional magnetic resonance imaging (fMRI) may be considered as a non-invasive alternative to the intracarotid amobarbital test^1–3^. However, the spatial pattern and strength of task-evoked activation are highly dependent on patient compliance and the specific language paradigm used^4,5^. Resting-state fMRI avoids this dependency on task performance, but prior studies showed that resting-state functional connectivity predicts task fMRI language dominance only modestly^6–9^.

We previously developed an approach to derive large-scale resting-state networks of the cerebral cortex^10^ as well as a multi-session hierarchical Bayesian model (MS-HBM) for estimating individual-specific networks with limited quantities of data – MS-HBM networks obtained using only 10 minutes of data were comparable to other approaches requiring approximately 50 minutes^11^. Large-scale functional networks vary across individuals^12–14^ and differ between healthy and pathological brains^15,16^. Moreover, individual-specific differences in network topography were able to predict inter-individual variation in cognition and personality^11^. Despite significant progress in estimating individual cortical networks in healthy individuals, their properties and behavioural relevance remain poorly characterized in drug-resistant epilepsy.

Here, we applied our approach for deriving group-average^10^ and individual-specific cortical networks^11^ to participants with drug-resistant temporal lobe and extra-temporal lobe epilepsy undergoing presurgical evaluation at the National Institute of Neurological Disorders and Stroke, National Institutes of Health (NIH dataset)^6^. We showed that individual-specific networks using MS-HBM trained on drug-resistant epilepsy were of higher quality than group-average networks and MS-HBM trained on healthy participants.

We further validated and tested generalizability of the MS-HBM model in an independent drug-resistant epilepsy dataset^17^, showing that during concurrent intracranial electrical stimulation with fMRI, cortical activation and deactivation better conformed to individual-specific network boundaries than to group-average boundaries. Finally, we showed that individual-specific networks predict task-based language dominance in individual patients.

Our model is publicly available (github link), which may be used to estimate high-quality individual-specific cortical networks and predict language dominance in a new individual with drug-resistant epilepsy using approximately 10 minutes of resting-state fMRI data.

## Methods

### Participants and Datasets

In this study, we included one open-source dataset of healthy participants and two independent datasets of participants with drug-resistant epilepsy undergoing pre-surgical evaluation. Human Connectome Project (HCP) participants^11^ had a mean age of 29.0 ± 4.0 years, 16 (40.0%) were male, and 12 (30.0%) self-identified as non-White and/or Hispanic. NIH epilepsy participants^6^ had a mean age of 31.9 ± 11.2 years and 33 (50.8%) were male. Of these, 42 (64.6%) had temporal lobe epilepsy and 23 (35.4%) had extra-temporal lobe epilepsy (Table S1). Electrical-stimulation fMRI (esfmri) dataset^17^ participants had a mean age of 35.8 ± 11.5 years, and 17 (65.4%) were male. Of these, 19 (73.1%) had temporal lobe epilepsy, 6 (23.1%) had extra-temporal lobe epilepsy, and 1 (3.8%) had undetermined seizure onset zone (Table S2).

All datasets included structural and resting-state fMRI scans. In the esfmri dataset, post-operative fMRI was acquired with concurrent intracranial electrical stimulation delivered through stereoencephalography electrodes and interleaved with echo planar imaging volume acquisition^17^. Alternating blocks of approximately 30 seconds of no stimulation or stimulation was delivered during the fMRI scan. No cognitive task was given to the participant, and no behavioural effects were evoked during the stimulation period.

**Figure 1:**
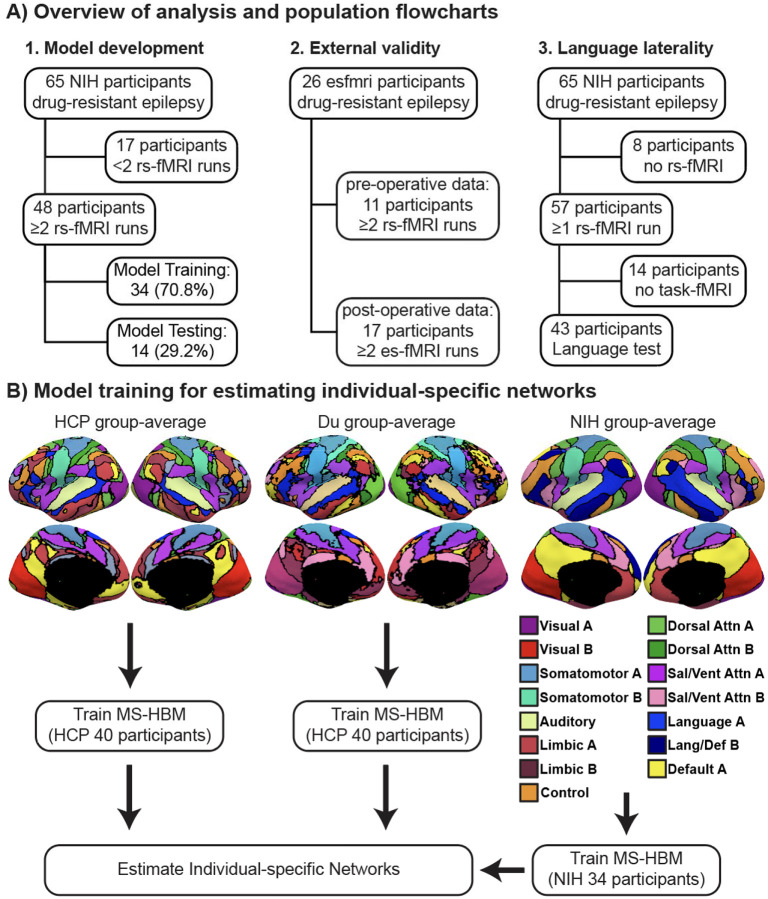
Study overview and population flow chart. (A) We developed a MS-HBM model for estimating individual-specific networks in drug-resistant epilepsy, validated and tested the model in an independent dataset, and showed that the topography of individual-specific language networks was able to predict task-based language dominance. (B) Group-average networks from healthy participants of the HCP dataset, the Du et al atlas, or drug-resistant epilepsy participants from the NIH dataset were used to train different MS-HBM models. 15-network labels were shown for NIH group-average networks. Networks in drug-resistant epilepsy appeared more focal compared to healthy participants

Further details regarding the demographics, data collection, and pre-processing are provided in the supplementary methods^6,17–19^. After pre-processing, there were 40 HCP participants with 4 runs each^11^. Of the 48 NIH participants with two or more runs, 34 (70.8%) participants were used for the training set, and 14 (29.2%) participants were used for the test set. For the esfmri dataset, there were 11 participants that had at least two valid pre-operative runs and 17 participants that had at least two valid post-operative runs (Figure 1A).

### Derivation of group-average networks and training of MS-HBM for estimating individual-specific networks

Our approach has been described previously^10,11^. At each cortical region (81,924 vertices), Pearson’s correlation between the fMRI time series and 1175 regions of interests were binarized by keeping the top 10% of correlations. The connectivity profiles were then clustered using a mixture of von Mises-Fisher distributions to derive the group-average 15-networks^10,20^. In addition to computing group-average networks for the 40 HCP participants and 34 NIH participants, we also compared our results to group-average networks derived from an independent dataset of 15 intensively-sampled healthy participants from Du et al^18^.

We trained MS-HBM models on either the 40 HCP participants (using the HCP group-average^11^ or Du group-average^18^) or 34 NIH participants to estimate model parameters, including the inter-subject functional connectivity variability, intra-subject functional connectivity variability, spatial smoothness prior, and the inter-subject spatial variability prior^11^. Individual-specific networks were visually confirmed using model-free seed-based functional connectivity. Inter-subject similarity in network topography across the 15-networks was quantified using the Dice similarity coefficient across all 57 NIH participants with at least one valid run.

### Validation and generalization of MS-HBM trained on drug-resistant epilepsy

To evaluate the quality of individual-specific networks estimated from MS-HBM trained on drug-resistant epilepsy or healthy participants, we computed two previously established metrics^11,14,21,22^: the weighted resting-state connectional homogeneity and weighted task (electrical-stimulation) functional inhomogeneity. These metrics encode the principle that if an individual-specific network captured the system-level organization of the individual’s cortex, each network should exhibit homogeneous connectivity and function.

We first evaluated the resting-state homogeneity of each network on the NIH test set of 14 participants that were not used for training MS-HBM. The generalizability of the MS-HBM model was then tested using pre-operative (11 participants) and post-operative (17 participants) esfmri data, as well as task inhomogeneity during electrical stimulation in the post-operative data. For estimating individual-specific networks in the post-operative data, we used only blocks with no stimulation after discarding the first 4 frames of each block to remove residual effects of the haemodynamic response from the previous block^23^.

To ensure that runs used for estimating individual-specific networks were independent from runs used for computing evaluation metrics, we used a leave-one-run-out cross-validation method. For each participant with n valid runs, individual-specific networks were estimated using n-1 runs, and evaluation metrics were computed on the left-out run. This was repeated for all runs, and an average result was obtained for each participant. Statistical tests were performed using two-tailed paired t-tests between each pair of networks (dof = number of participants – 1). Statistical significance was taken to be q<0.05 after false discovery rate correction.

The average resting-state homogeneity and task inhomogeneity were compared across six networks: (1) HCP group-average networks; (2) individual-specific networks estimated using HCP MS-HBM; (3) Du group-average networks; (4) individual-specific networks estimated using Du MS-HBM; (5) NIH group-average networks; and (6) individual-specific networks estimated using NIH MS-HBM. Resting-state homogeneity was computed by averaging the Pearson’s correlations between each vertex’s time course with the average time course of its network. The correlations were then averaged across all 15 networks while accounting for network size^11,21,22^. Task inhomogeneity was computed as the average standard deviation of cortical activation z-scores within each network from unthresholded generalized linear models of intracranial electrical stimulation^19^. The standard deviation was then averaged across all 15 networks while accounting for network size^11,21,22^. A higher standard deviation indicated higher task inhomogeneity. Further details on the computation of the generalized linear models are provided in the supplementary methods.

To visualise the effects of intracranial electrical stimulation on cortical networks, group-average network boundaries and individual-specific network boundaries were overlayed on the electrical-stimulation z-score activation maps. We hypothesised that electrical-stimulation evoked activity would align more closely to individual-specific network boundaries than group-average network boundaries.

### Predicting language dominance using individual-specific network topography

All patients underwent language task fMRI using an auditory description decision task as described previously^5,6^. Task-based language dominance was determined by a trained epileptologist (SI) through visual inspection of activation maps in the frontal and temporal regions at three thresholds (p < .01, p < .001, top 10% of activations) and confirmed at a multi-disciplinary epilepsy conference. There were a total of 43 NIH participants with valid task language dominance and at least one valid resting-state fMRI run.

In several participants with drug-resistant epilepsy, regions typically associated with the language network exhibited connectivity patterns characteristic of the default mode network, suggesting network reorganization. To capture this variability, we computed a resting-state laterality index (LI) based on the network topography of both the Language A and Language/ Default B networks:

$$\text{Resting-state LI}=\frac{LH-RH}{LH+RH}\text{,}$$

where LH and RH represent the number of vertices in the left and right hemispheres, respectively, assigned to the individual-specific Language A and Language/ Default B networks. Resting-state LI values for participants with left, bilateral, or right task-based language dominance were compared using one-way ANOVA, with p<0.05 considered statistically significant. Finally, receiver operating curves and area-under-the-curve statistics were computed to evaluate the predictive power of resting-state LI for each language dominance group.

## Results

### Individual-specific resting-state networks in drug-resistant epilepsy capture inter-individual variability and outperform group-level networks

We estimated large-scale resting-state networks in drug-resistant epilepsy despite structural and functional abnormalities. Networks were categorized into 9 canonical groups (Visual, Somatomotor, Auditory, Limbic, Control, Dorsal Attention, Salience/Ventral Attention, Language, and Default), which were broadly consistent with prior literature^10,11,18,24^. Group-level networks in healthy participants were more spatially distributed than those in drug-resistant epilepsy (Figure 1B). For example, association networks such as the control network were subdivided into three networks in HCP data^11^, but were represented by a single, less distributed network in drug-resistant epilepsy. Instead, two spatially focal limbic networks emerged in orbitofrontal and parahippocampal regions. These differences were not explained by motion as the average relative root mean square framewise displacement was 0.059 ± 0.023 vs. 0.076 ± 0.015 and the average voxel-wise differentiated signal variance (DVARS)^25^ was 23.0 ± 4.6 vs. 58.4 ± 6.8 for the NIH dataset compared to the HCP dataset respectively.

**Figure 2:**
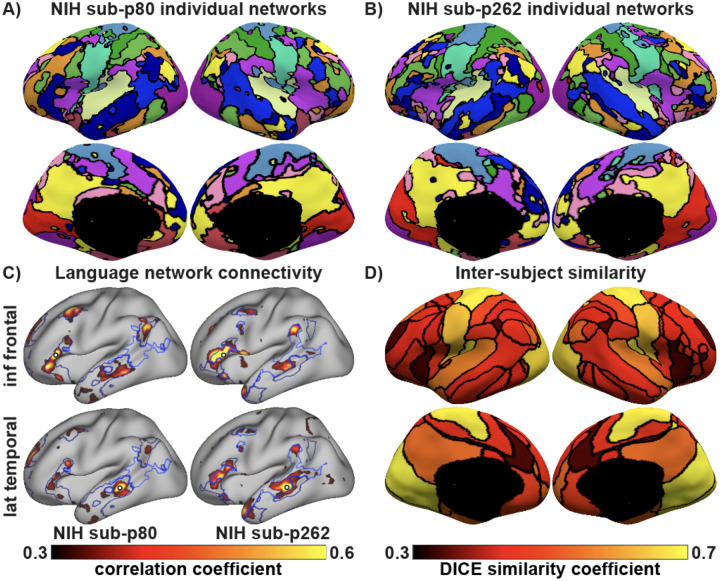
Individual-specific resting-state cortical networks in drug-resistant epilepsy. (A-B) Individual-specific networks of two representative NIH participants estimated using NIH MS-HBM. (C) Language network boundaries of both participants showing functional connectivity from identical seeds placed in the inferior frontal and lateral temporal region to different language networks in the two subjects. (D) Dice similarity coefficient for the 15-networks across all 57 NIH participants. Higher dice coefficients (yellow) indicate greater inter-subject topographic similarity, while lower dice coefficients (dark red) indicate higher variability. Inter-subject variability in drug-resistant epilepsy is higher in association than sensory networks.

Using MS-HBM, individual-specific networks captured inter-individual variability while preserving canonical topographical features of healthy participants. However, in some participants, inferior frontal language regions exhibited stronger connectivity with middle temporal and temporopolar areas typically associated with the default mode network in healthy individuals, suggesting network reorganization (Figure 2A vs. 2B). Language connectivity was visually confirmed using identical seeds in the inferior frontal and lateral temporal regions across participants (Figure 2C). Finally, we observed that somatomotor and visual networks exhibited lower inter-subject variability (DICE similarity coefficient ranging from 0.66 – 0.71) than association networks such as the salience/ ventral attention, dorsal attention, control, and language networks (DICE similarity coefficient ranging from 0.35 – 0.50; Figure 2D).

**Figure 3:**
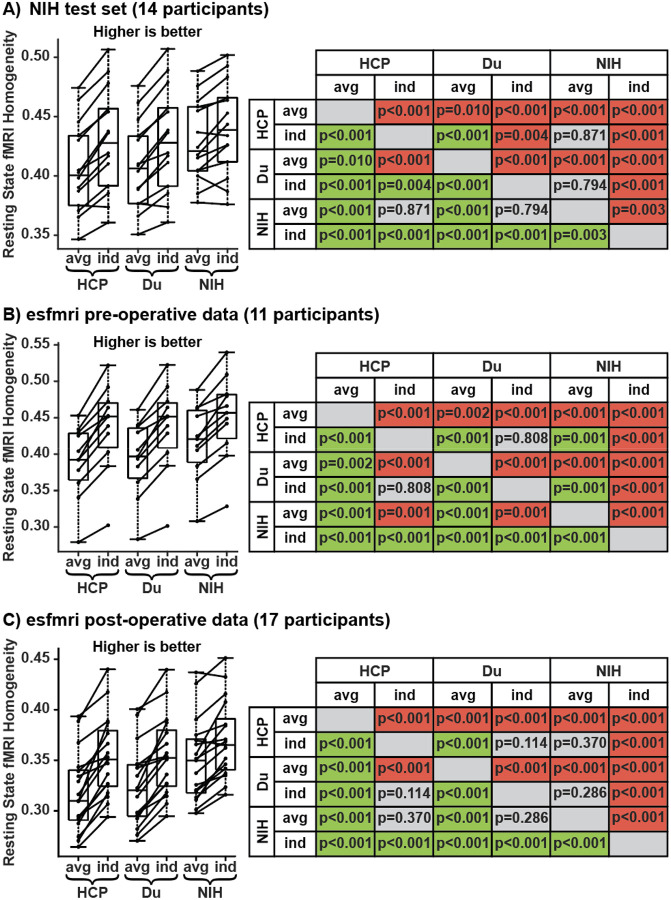
Resting-state homogeneity of group-average and individual-specific networks. Individual-specific networks estimated using NIH MS-HBM outperformed group-average networks or MS-HBM trained on healthy participants. Results were consistent across the (A) NIH test set, (B) esfmri pre-operative data, and (C) esfmri post-operative data. Six different networks were evaluated: (1) group-average and (2) individual-specific networks estimated from the HCP data; (3) group-average and (4) individual-specific networks estimated from the Du atlas; (5) group-average and (6) individual-specific networks estimated from the NIH data. Left: boxplots of median resting-state homogeneity. Right: t-test p-values comparing pairs of approaches. Green indicates that the approach on the row performed significantly better than the column, red indicates worse, and grey indicates non-significant differences, after correcting for multiple comparisons using false discovery rate of q < 0.05.

Next, we compared the quality of group-level networks estimated from the HCP data, Du atlas, and NIH data with the individual-specific networks estimated in 14 NIH subjects using the three MS-HBM models. Individual-specific networks estimated using NIH MS-HBM performed significantly better than all group-average networks and MS-HBM trained on healthy participants, even after correcting for multiple comparisons (Figure 3A).

### MS-HBM trained on drug-resistant epilepsy generalized to an independent dataset and showed the lowest electrical-stimulation inhomogeneity

MS-HBM trained on healthy participants might perform worse than NIH MS-HBM because of site and scanner differences. Therefore, we tested if NIH MS-HBM was generalizable to an independent dataset of drug-resistant epilepsy. Individual-specific networks estimated using NIH MS-HBM performed significantly better in resting-state homogeneity than all group-average networks and MS-HBM trained on healthy participants, even after correcting for multiple comparisons (Figure 3B and 3C).

**Figure 4:**
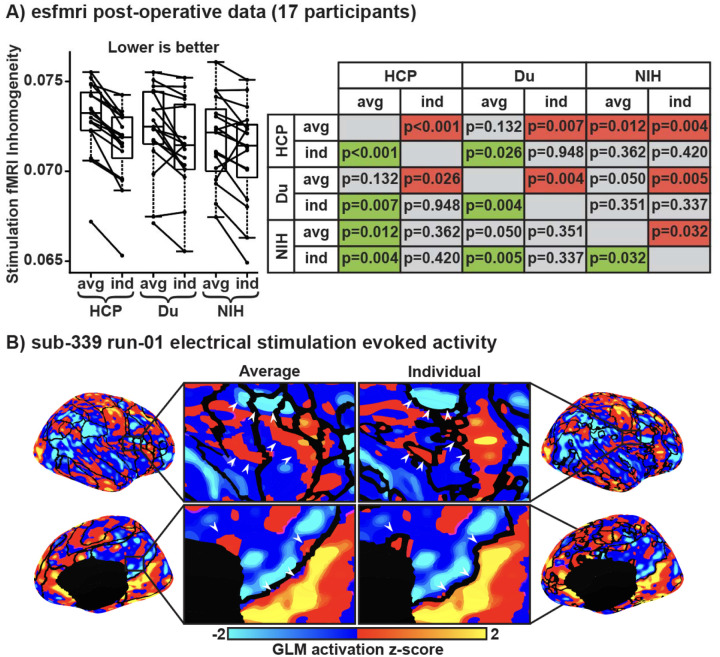
Electrical-stimulation inhomogeneity and evoked cortical activation map. Individual-specific networks estimated using NIH MS-HBM performed the best in electrical-stimulation inhomogeneity and network boundaries aligned better to electrical-stimulation evoked activity compared to group-average networks. (A) Left shows boxplots of median electrical stimulation inhomogeneity across the six networks: (1) group-average and (2) individual-specific networks estimated from the HCP data; (3) group-average and (4) individual-specific networks estimated from the Du atlas; (5) group-average and (6) individual-specific networks estimated from the NIH data. Right: t-test p-values comparing pairs of approaches. Green indicates that the approach on the row performed significantly better than the column, red indicates worse, and grey indicates non-significant differences, after correcting for multiple comparisons using false discovery rate of q < 0.05. (B) Generalised linear model of electrical-stimulation during fMRI showing z-score normalised beta-coefficients overlayed with the NIH group-average network boundaries on the left and the participant’s individual-specific network boundaries on the right. White arrowheads show evoked activity that aligned better with individual-specific networks than group-average networks.

In addition, individual-specific networks estimated using NIH MS-HBM performed significantly better than group-average networks for electrical-stimulation inhomogeneity, even after correcting for multiple comparisons (Figure 4A). We observed that during concurrent intracranial electrical stimulation and fMRI scans, cortical evoked activity aligned better to individual-specific network boundaries than group-average network boundaries (Figure 4B). Taken together, these results suggest that MS-HBM trained using drug-resistant epilepsy can estimate high-quality, generalizable individual-specific networks that captured system-level cortical organization better than group-average networks.

### Individual-specific language network topography predicts task-based language dominance

Having shown that individual-specific networks estimated from NIH MS-HBM captured inter-individual variability in cortical resting-state networks, we next tested if language network topography were able to predict task-based language dominance. Resting-state LI differed significantly across left (mean LI = 0.165 ± 0.106), bilateral (mean LI = 0.056 ± 0.074), and right (mean LI = 0.023 ± 0.055) task-based language dominance (p = 0.002). Receiver operating curve analyses showed area-under-the-curve statistics of 0.82 for left, 0.72 for bilateral, and 0.83 for right language dominance versus other participants. Conversely, individual-specific networks estimated using MS-HBM trained on healthy participants were not able to differentiate between task-based language dominance (HCP MS-HBM: p = 0.150; Du MS-HBM: p = 0.303).

## Discussion

Precision functional mapping techniques have been shown to reliably estimate the intrinsic cortical organisation of an individual, but typically required long scan durations of an hour or more fMRI data^12,13,26^ and have not been applied to drug-resistant epilepsy. Using MS-HBM, we showed that despite structural abnormalities and differences in functional connectivity, individual-specific networks may be reliably estimated in drug-resistant epilepsy even with only approximately 10 minutes of single-session resting-state fMRI data. MS-HBM trained on drug-resistant epilepsy captured the organization of an individuals’ cerebral cortex better than group-average networks or MS-HBM trained on healthy participants. Furthermore, NIH MS-HBM generalized to an external dataset of drug-resistant epilepsy despite site, scanner, and pre-processing differences.

Although cortical networks in drug-resistant epilepsy broadly resembled canonical resting-state networks identified in healthy participants, we observed that spatial topography was more focal and individual-level differences emerged that were not typically observed in healthy participants. These differences could not be explained by motion. In particular, inferior frontal language regions exhibited stronger connectivity with regions typically associated with the default mode network in healthy individuals, which may be due to the long-term effects of drug-resistant epilepsy on functional connectivity^27–32^. Existing data showed that alterations in functional connectivity appear to be related to the duration and severity of epilepsy, and were associated with cognitive impairment, suggesting progressive cortical reorganization in the setting of recurrent seizures over time^33,34^. Thus, further research using precision functional mapping may provide deeper insight into the effects of chronic epileptogenic activity on reorganization of the resting-state cortical networks^35^.

These differences in the resting-state language network in drug-resistant epilepsy may potentially explain why previous research in resting-state laterality using seed-based connectivity were limited in predicting task-based language dominance^6–9^. Here, we showed that by accounting for both the Language A and the Language/ Default B networks, individual-specific network topography was able to predict task-based language dominance. Future studies should validate this resting-state laterality index against the intracarotid amobarbital test^3^ or cortical electrical stimulation^36^ and assess generalizability across other drug-resistant epilepsy cohorts. By characterizing functional organization at the individual level, we hypothesize that individual-specific networks may also predict the lateralization of other cognitive functions^33,37^ as well as post-operative functional outcomes^38–40^. Thus, mapping individual-specific cortical networks from brief resting-state scans may offer a practical, non-invasive approach for presurgical epilepsy evaluation in the future^41^.

## Methodological Considerations

We used healthy participants from the Human Connectome Project and two drug-resistant epilepsy datasets to show that MS-HBM trained on drug-resistant epilepsy outperformed group-average networks and MS-HBM trained on healthy participants despite site, scanner, and pre-processing differences. We studied participants with temporal lobe and extra-temporal lobe epilepsy undergoing presurgical evaluation, and further validation of the networks in other types of drug-resistant epilepsy (e.g. idiopathic generalized epilepsy) is required.

The esfmri dataset contained unique data that allowed us to validate the quality of individual-specific networks using evoked activity during concurrent intracranial electrical stimulation. However, it must be noted that the esfmri dataset did not contain true post-operative resting-state fMRI data and we concatenated blocks of fMRI data when no stimulation was applied to simulate resting-state fMRI scans^17^. When comparing functional connectivity within the same participants across pre-operative and post-operative data, we identified differences that may reflect either (1) the effects of neurosurgery; (2) distortion in the fMRI signal due to the implanted electrodes; or (3) scanner differences. Thus, we tested the quality of individual-specific networks estimated using both the pre-operative and post-operative data and showed that individual-specific networks estimated by NIH MS-HBM performed the best regardless of the data used.

## Conclusion

We present a method for estimating reliable, high-quality individual-specific cortical networks in drug-resistant epilepsy and showed that individual-specific language network topography predicts task-based language dominance. Our model is publicly available (github link), which may be used to estimate individual-specific cortical networks and predict language dominance in a new individual with drug-resistant epilepsy given approximately 10 minutes of single-session resting-state fMRI data.

## Supplementary Material

1

## Figures and Tables

**Figure 5:**
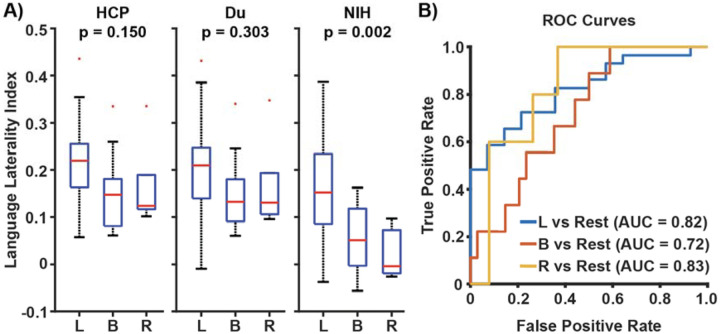
Individual-specific language network topography predicts task-based language dominance. (A) Boxplots of language laterality index for participants with left, bilateral, or right language dominance derived from task fMRI, comparing individual-specific networks estimated from HCP MS-HBM, Du MS-HBM, or NIH MS-HBM. (B) Receiver operating curves and area-under-the-curve statistic for predicting language dominance using individual-specific networks estimated by NIH MS-HBM.

## Key Points

### Question:

Can individual-specific network topography from resting-state functional magnetic resonance imaging (fMRI) predict task-based language dominance in drug-resistant epilepsy?

### Findings:

In this multi-centre case-control study of 91 participants with drug-resistant epilepsy and 40 healthy controls, individual-specific networks outperformed group-average networks and generalized well to an independent cohort. Language network topography differed significantly across left (mean lateralization index (LI) = 0.165 ± 0.106), bilateral (LI = 0.056 ± 0.074), and right (LI = 0.023 ± 0.055) dominance groups (p = 0.002).

### Meaning:

Resting-state fMRI can estimate high-quality individual-specific cortical networks that predict language dominance, providing a non-invasive tool for presurgical evaluation.
