# Supplementary material for: Individual-specific resting-state networks predict language dominance in drug-resistant epilepsy
Source: medRxiv. 2025 Nov 25:2025.11.21.25340716. Preprint. [Version 1] doi: 10.1101/2025.11.21.25340716 (PMC12676542; doi:10.1101/2025.11.21.25340716)
Supplement: 1 [file NIHPP2025.11.21.25340716V1-supplement-1.pdf]

medRxiv preprint doi: https://doi.org/10.1101/2025.11.21.25340716; this version posted November 25, 2025. The copyright holder for this preprint (which was not certified by peer review) is the author/funder, who has granted medRxiv a license to display the preprint in perpetuity.

It is made available under a CC-BY 4.0 International license .

Supplementary Methods

Participants and Datasets

The HCP dataset consisted of 40 participants from the Human Connectome Project (HCP) S900 release. The mean age was 29.0 ± 4.0 years, there were 16 (40.0%) male participants, and 12 (30.0%) participants self-reported as non-White and/or Hispanic race. Each subject had one 0.7mm isotropic T1 structural scan and four runs of 2mm isotropic resting-state fMRI with a TR of 0.72s and lasting 14min 33s each. All imaging data were collected on a custom-made Siemens 3 T Skyra scanner using a multiband sequence. Details of the data collection may be found elsewhere[1,2].

The NIH dataset consisted of 65 participants with drug-resistant epilepsy undergoing presurgical evaluation at the NIH epilepsy center[3] (Table S1). The mean age of participants was 31.9 ± 11.2 years, and there were 33 (50.8%) male participants. There were 42 (64.6%) participants with temporal lobe epilepsy, and 23 (35.4%) participants with extra-temporal lobe epilepsy. All participants gave informed written consent under a research protocol approved by the NIH Combined Neurosciences Institutional Review Board[3]. Each participant had one 0.8 x 0.75 x 0.75mm T1 and T2 structural scans, up to four runs of 3mm isotropic resting-state fMRI with a TR of 2.5s and lasting 6min 25s each, and a 3mm isotropic auditory description decision language task fMRI run with a TR of 2.0s and lasting 5min. All imaging data were collected on a 3.0 Tesla scanner at the NIH Nuclear Magnetic Resonance Center using a multiband sequence on either a Siemens Skyra scanner or a GE Discovery scanner. Details of the data collection may be found elsewhere[3].

The esfmri dataset consisted of 26 participants with drug-resistant epilepsy undergoing presurgical evaluation at the University of Iowa[4] (Table S2). The mean age of participants was 35.8 ± 11.5 years, and there were 17 (65.4%) male participants. There were 19 (73.1%) participants with temporal lobe epilepsy, 6 (23.1%) participants with extra-temporal lobe

medRxiv preprint doi: https://doi.org/10.1101/2025.11.21.25340716; this version posted November 25, 2025. The copyright holder for this preprint (which was not certified by peer review) is the author/funder, who has granted medRxiv a license to display the preprint in perpetuity.

It is made available under a CC-BY 4.0 International license .

epilepsy, and 1 (3.8%) participant where the seizure onset zone was not determined. Intracranial electrodes were implanted to localize the epileptogenic zone, and were determined by clinical criteria. All participants gave informed written consent under a research protocol approved by the University of Iowa Institutional Review Board[4]. Each participant had one 1.0 x 1.0 x 0.8mm T1 structural scan, up to five runs of 3.4 x 3.4 x 4.0mm pre-operative resting state fMRI with a TR of 2.26s lasting 4.8min each, and up to eleven runs of 3mm isotropic electrical-stimulation fMRI with a TR of 3.0s lasting approximately 10min each. For each post-operative fMRI scan, bipolar intracranial electrical stimulation using depth electrodes was interleaved between echo planar imaging volume acquisition during a 100ms period where there was no scanner radiofrequency or gradient switching[4]. Stimulation was blocked with alternating durations of approximately 30 seconds of STIM-ON and STIM-OFF each. No cognitive task was given to the participant, and no behavioural effects were evoked during the stimulation period. Details of the data collection may be found elsewhere[4,5].

No.	Age	Sex	Hand	Seizure Onset Zone	Task Laterality	MRI	Valid Runs
1	26-30	F	R	Right Mesial Temporal Sclerosis	L	Siemens Skyra	2
2	20-25	F	R	Right Parietal	U	GE Discovery MR750	2
3	36-40	M	R	Left > Right Temporal	B	GE Discovery MR750	1
4	30-35	M	R	Right > Left Medial Temporal	L	Siemens Skyra	2
5	20-25	F	R	Left Mesial Temporal Sclerosis	U	GE Discovery MR750	1
6	36-40	M	R	Right Mesial Temporal Sclerosis	L	Siemens Skyra	0
7	30-35	F	R	Right Temporoparietal	L	GE Discovery MR750	2
8	26-30	F	R	Right Temporal	B	GE Discovery MR750	2
9	30-35	F	R	Right Mesial Temporal Sclerosis	L	Siemens Skyra	3
10	20-25	M	L	Left Mesial Temporal Sclerosis	L	GE Discovery MR750	2

medRxiv preprint doi: https://doi.org/10.1101/2025.11.21.25340716; this version posted November 25, 2025. The copyright holder for this preprint (which was not certified by peer review) is the author/funder, who has granted medRxiv a license to display the preprint in perpetuity.

It is made available under a CC-BY 4.0 International license .

11	36-40	F	R	Left Temporal	R	GE Discovery MR750	2
12	56-60	F	R	Right Frontal	L	GE Discovery MR750	2
13	16-20	M	R	Right Frontal	L	GE Discovery MR750	1
14	36-40	F	R	Left Frontal/ Insula	U	GE Discovery MR750	1
15	20-25	M	R	Right Frontal	U	GE Discovery MR750	2
16	30-35	M	R	Left Temporal Periventricular Nodular Heterotopia	B	Siemens Skyra	3
17	36-40	M	L	Left > Right Frontal	U	GE Discovery MR750	1
18	36-40	F	L	Left Mesial Temporal Sclerosis	R	GE Discovery MR750	2
19	20-25	M	R	Left Frontal/ Insula	L	GE Discovery MR750	2
20	36-40	M	R	Right Frontal	U	GE Discovery MR750	2
21	30-35	F	R	Left Mesial Temporal Sclerosis	L	Siemens Skyra	1
22	16-20	F	R	Left Frontotemporal	R	GE Discovery MR750	2
23	51-55	M	R	Left Temporal	L	Siemens Skyra	3
24	26-30	F	R	Right Temporal	R	GE Discovery MR750	2
25	30-35	F	R	Left Temporal	L	Siemens Skyra	2
26	16-20	F	R	Right Frontal	L	GE Discovery MR750	2
27	36-40	F	R	Left Temporal	B	GE Discovery MR750	0
28	56-60	F	R	Left Mesial Temporal Sclerosis	L	Siemens Skyra	3
29	26-30	M	B	Bilateral Frontal	L	GE Discovery MR750	2
30	30-35	F	R	Left > Right Temporal	L	Siemens Skyra	3
31	26-30	F	L	R Insula > Frontal	R	GE Discovery MR750	2
32	20-25	F	B	Right Polymicrogyria, Bilateral Periventricular Nodular Heterotopia	B	GE Discovery MR750	2
33	51-55	M	R	Left Frontal Schizencephaly	U	GE Discovery MR750	0
34	26-30	F	R	Left Mesial Temporal Sclerosis	B	Siemens Skyra	3
35	41-45	M	R	Right Frontal	L	Siemens Skyra	3

medRxiv preprint doi: https://doi.org/10.1101/2025.11.21.25340716; this version posted November 25, 2025. The copyright holder for this preprint (which was not certified by peer review) is the author/funder, who has granted medRxiv a license to display the preprint in perpetuity.

It is made available under a CC-BY 4.0 International license .

36	26-30	F	R	Right Mesial Temporal Sclerosis	U	Siemens Skyra	1
37	30-35	M	R	Right Parietal	L	Siemens Skyra	3
38	41-45	F	L	Right Frontal	B	Siemens Skyra	3
39	16-20	F	R	Right Temporal	U	Siemens Skyra	0
40	56-60	M	R	Left Temporal	B	Siemens Skyra	1
41	20-25	M	R	Left Temporal	L	Siemens Skyra	3
42	36-40	F	R	Right Temporal	U	Siemens Skyra	3
43	20-25	M	L	Right Temporal Arteriovenous Malformation	L	Siemens Skyra	3
44	16-20	M	L	Left Parietal	B	Siemens Skyra	3
45	20-25	M	R	Left Frontal	L	Siemens Skyra	2
46	26-30	M	R	Left Temporal Low Grade Glioma	L	Siemens Skyra	3
47	20-25	F	R	Left Frontal	L	Siemens Skyra	3
48	26-30	M	R	Right Parieto-occipital	L	Siemens Skyra	0
49	30-35	M	R	Left Mesial Temporal Sclerosis	U	Siemens Skyra	3
50	20-25	M	R	Right Frontal	L	Siemens Skyra	4
51	30-35	M	L	Left Temporal	U	Siemens Skyra	3
52	20-25	F	R	Right Temporal	L	Siemens Skyra	2
53	26-30	F	R	Left Temporal	U	Siemens Skyra	3
54	56-60	M	R	Right Temporal	L	Siemens Skyra	1
55	6-10	M	R	Left Frontal	L	Siemens Skyra	3
56	26-30	M	R	Bilateral Temporal	U	Siemens Skyra	3
57	36-40	M	R	Left Temporal	U	Siemens Skyra	3
58	20-25	M	R	Right Temporal	L	Siemens Skyra	3
59	51-55	F	R	Right Temporal	L	Siemens Skyra	3
60	30-35	F	R	Right Temporal	R	Siemens Skyra	0
61	20-25	F	R	Right Parietal/ Temporal	L	Siemens Skyra	0
62	26-30	M	R	Left Temporal	U	Siemens Skyra	3
63	26-30	M	R	Right Temporal	L	Siemens Skyra	2
64	51-55	F	R	Left Temporal	L	Siemens Skyra	0
65	30-35	M	R	Left Temporal	B	Siemens Skyra	2

Table S1: Demographic, MRI, and epilepsy characteristics of participants from the NIH dataset.

medRxiv preprint doi: https://doi.org/10.1101/2025.11.21.25340716; this version posted November 25, 2025. The copyright holder for this preprint (which was not certified by peer review) is the author/funder, who has granted medRxiv a license to display the preprint in perpetuity.

It is made available under a CC-BY 4.0 International license .

No.	Age	Sex	Hand	Seizure Onset Zone	pre-op MRI	pre-op Runs	post-op MRI	post-op Runs
1	46-50	F	L	Left mesial temporal lobe, Left frontal lobe	Siemens Trio	0	Siemens Trio	5
2	31-35	M	R	Right anterior frontal lobe	Siemens Trio	0	Siemens Trio	2
3	46-50	F	R	Left mesial temporal lobe	Siemens Trio	0	Siemens Skyra	1
4	31-35	F	R	Right mesial temporal lobe, Left mesial temporal encephalomalacia	Siemens Trio	1	Siemens Skyra	4
5	26-30	M	R	Left insula	Siemens Trio	1	Siemens Skyra	1
6	26-30	F	R	Bilateral mesial temporal lobe	Siemens Trio	5	Siemens Skyra	0
7	31-35	F	R	Right mesial temporal lobe	Siemens Trio	4	Siemens Skyra	7
8	46-50	F	R	Right hippocampus	Siemens Trio	0	Siemens Skyra	0
9	41-45	M	L	Left occipital lobe	Siemens Trio	5	Siemens Skyra	4
10	31-35	M	R	Left mesial temporal lobe	Siemens Trio	1	Siemens Skyra	5
11	36-40	M	L	Right temporal pole, Left temporal base	Siemens Trio	5	Siemens Skyra	10
12	31-35	M	R	Bilateral mesial temporal lobe	Siemens Trio	3	Siemens Skyra	2
13	41-45	M	R	Not determined	None	-	Siemens Skyra	6
14	31-35	M	B	Left frontal cystic mass	GE Discovery	2	Siemens Skyra	0
15	36-40	M	R	Left mesial temporal lobe	None	-	Siemens Skyra	1
16	26-30	M	R	Right mesial temporal lobe	None	-	Siemens Skyra	8
17	31-35	M	R	Left temporal pole	GE Discovery	2	Siemens Skyra	0
18	46-50	F	R	Right mesial temporal lobe	GE Discovery	3	Siemens Skyra	2
19	36-40	M	R	Right mesial temporal lobe, Right frontal pole	GE Discovery	2	Siemens Skyra	0

medRxiv preprint doi: https://doi.org/10.1101/2025.11.21.25340716; this version posted November 25, 2025. The copyright holder for this preprint (which was not certified by peer review) is the author/funder, who has granted medRxiv a license to display the preprint in perpetuity.

It is made available under a CC-BY 4.0 International license .

20	21-25	M	R	Right amygdala	None	-	Siemens Skyra	7
21	11-15	M	R	Left superior frontal gyrus cavernoma	None	-	Siemens Skyra	3
22	21-25	F	R	Right mesial temporal lobe, Possible right frontal base	GE Discovery	3	Siemens Skyra	6
23	56-60	M	R	Left mesial temporal lobe	GE Discovery	1	Siemens Skyra	2
24	56-60	F	R	Left mesial temporal lobe	None	-	Siemens Skyra	0
25	16-20	M	R	Left frontal encephalomalacia	GE Discovery	4	Siemens Skyra	3
26	21-25	M	L	Right mesial temporal lobe	GE Discovery	5	Siemens Skyra	2

Table S2: Demographic, MRI, and epilepsy characteristics of participants from the esfmri dataset.

Pre-processing of Imaging Data

The HCP dataset was released with data already pre-processed (smoothed using a 2mm full-width half-maximum kernel, denoised with ICA-FIX and aligned with MSMAll) and were detailed elsewhere[6,7]. The preprocessed fMRI data was projected onto the FreeSurfer fsaverage6 surface space. Additional nuisance regression, censoring, and spatial smoothing was computed using the surface-based pipeline of Yeo et al[6].

Pre-processing of the NIH dataset followed the surface-based pipeline of Yeo et al[8,9]. Structural data were processed using recon-all (FreeSurfer 7.1.1)[10]. Resting-state fMRI data were processed with the following: (i) removal of first 4 frames, (ii) slice time correction using FSL[11], (iii) motion correction and outlier detection using rigid body translation and rotation[11], (iv) susceptibility-induced spatial distortion correction[12], (v) multi-echo denoising[13], (vi) boundary-based registration[14], (vii) regression of the global, white matter, ventricular signals,

medRxiv preprint doi: https://doi.org/10.1101/2025.11.21.25340716; this version posted November 25, 2025. The copyright holder for this preprint (which was not certified by peer review) is the author/funder, who has granted medRxiv a license to display the preprint in perpetuity.

It is made available under a CC-BY 4.0 International license .

6 motion parameters, and their temporal derivatives, (viii) censored frames were interpolated with the Lomb-Scargle periodogram[15], (ix) bandpass filtering of 0.009Hz – 0.08Hz, and lastly, (x) the data was projected onto FreeSurfer fsaverage6 surface space and smoothed using a 6mm full-width half-maximum kernel. Outlier detection using framewise displacement (FD) and voxel-wise differentiated signal variance (DVARS) were computed using fsl_motion_outliers[16]. Frames that exceeded a threshold of 0.2mm FD or 50 DVARS as well as one frame before and two frames after were censored. Segments of data lasting less than five contiguous frames were also censored[17]. After pre-processing, 8 participants did not have more than one valid resting-state run, 9 participants had one run, 23 participants had two runs, 24 participants had three runs, and one participant has four runs.

The esfmri dataset was released with data pre-processed using fMRIPrep 1.5.1rc1[18] (based on Nipype 1.2.0[19]) and was described in detail elsewhere[4]. Frames that exceeded a threshold of 0.2mm FD or 1.2 standardized derivative of the root-mean-square variance were censored. Runs that had more than 50% motion outliers were discarded. Linear regression was used to regress out 6 motion parameters, the whole brain, white matter, and cerebrospinal fluid regressors and their derivatives. The fMRI time-series were then resampled to the FreeSurfer fsaverage6 space and spatially smoothed using a 6mm full-width half-maximum kernel (FreeSurfer 5.3.0)[10]. After pre-processing, there were 11 participants that had at least two valid pre-operative runs and 17 participants that had at least two valid post-operative runs.

Derivation of group-average networks and training of MS-HBM for estimating individual-specific networks

Our approach has been described previously[6,8]. Briefly, the pre-processed fMRI data were projected onto fsaverage6 surface meshes that consisted of 40,962 vertices per hemisphere. The connectivity profile of a cortical region (vertex) was defined to be its functional coupling

medRxiv preprint doi: https://doi.org/10.1101/2025.11.21.25340716; this version posted November 25, 2025. The copyright holder for this preprint (which was not certified by peer review) is the author/funder, who has granted medRxiv a license to display the preprint in perpetuity.

It is made available under a CC-BY 4.0 International license .

to 1175 regions of interest (ROIs), which consisted of single vertices uniformly distributed across the fsaverage6 surface meshes. For each run, the Pearson's correlation between the fMRI time series at each spatial location (81,924 vertices) and the 1175 ROIs were computed. The 81,924 x 1175 correlation matrix was then binarized by keeping the top 10% of correlations. To obtain group-average networks, each vertex's connectivity profiles were averaged across all resting-state fMRI runs of all participants. The averaged connectivity profiles were then clustered using a mixture of von Mises-Fisher distributions to obtain the 15-network group-average for each dataset[8,20].

We developed group-average networks and trained MS-HBM using either 40 participants from the Human Connectome Project (HCP dataset)[6], or 34 participants with drug-resistant epilepsy from the National Institutes of Health (NIH dataset). In addition, we used a group-average atlas of 15 densely sampled healthy participants from Du et al[7] to train MS-HBM on the 40 participants from the HCP dataset. Model parameters estimated included the inter-subject functional connectivity variability, intra-subject functional connectivity variability, spatial smoothness prior, and the inter-subject spatial variability prior[6]. Using each of the three MS-HBM models, individual-specific networks were estimated for each participant with drug-resistant epilepsy. For all individual-specific networks estimated, the weight of the group spatial prior was taken to be 80, and the weight of the smoothness prior was taken to be 10[6].

Generalized linear models of intracranial electrical stimulation during fMRI scans

Generalized linear models were used to quantify the effects of electrical stimulation on cortical activity. A boxcar function of the stimulus duration (varies from 50-90 ms) for each post-operative run was computed[4]. The hemodynamic response function was then modelled by convolving the boxcar function with an optimised set of three gamma basis functions generated using FSL Linear Optimal Basis Sets[21] to account for any non-canonical

medRxiv preprint doi: https://doi.org/10.1101/2025.11.21.25340716; this version posted November 25, 2025. The copyright holder for this preprint (which was not certified by peer review) is the author/funder, who has granted medRxiv a license to display the preprint in perpetuity.

It is made available under a CC-BY 4.0 International license .

haemodynamic response function shapes caused by the stimulation (parameters used were m1 = 0-2s; m2 = 3-6s; m3 = 3-8s; m4 = 3-8s; c = 0-0.3). The amplitude of all basis functions were normalized to peak values of 1 by dividing by the maximum amplitude before computing the haemodynamic response function.

The generalized linear models were computed using freesurfer's mri_glmfit on the fsaverage6 data and corrected at the vertex-level for the lag one autoregression correlation matrix averaged across all valid runs. The vertex-level unthresholded beta-coefficients of the contrast consisting of all three haemodynamic response function shapes were then Z-score normalised by subtracting the mean and dividing by the standard deviation across all beta-coefficients of both hemispheres.

medRxiv preprint doi: https://doi.org/10.1101/2025.11.21.25340716; this version posted November 25, 2025. The copyright holder for this preprint (which was not certified by peer review) is the author/funder, who has granted medRxiv a license to display the preprint in perpetuity.

It is made available under a CC-BY 4.0 International license .

References

1. Van Essen DC, Ugurbil K, Auerbach E, et al. The Human Connectome Project: a data acquisition perspective. *Neuroimage*. Oct 1 2012;62(4):2222-31. doi:10.1016/j.neuroimage.2012.02.018

2. Smith SM, Beckmann CF, Andersson J, et al. Resting-state fMRI in the Human Connectome Project. *Neuroimage*. Oct 15 2013;80:144-68. doi:10.1016/j.neuroimage.2013.05.039

3. Rolinski R, You X, Gonzalez‐Castillo J, et al. Language lateralization from task‐based and resting state functional MRI in patients with epilepsy. *Human Brain Mapping*. 2020;41(11):3133-3146. doi:10.1002/hbm.25003

4. Thompson WH, Nair R, Oya H, et al. A data resource from concurrent intracranial stimulation and functional MRI of the human brain. *Sci Data*. Aug 5 2020;7(1):258. doi:10.1038/s41597-020-00595-y

5. Oya H, Howard MA, Magnotta VA, et al. Mapping effective connectivity in the human brain with concurrent intracranial electrical stimulation and BOLD-fMRI. *J Neurosci Methods*. Feb 1 2017;277:101-112. doi:10.1016/j.jneumeth.2016.12.014

6. Kong R, Li J, Orban C, et al. Spatial Topography of Individual-Specific Cortical Networks Predicts Human Cognition, Personality, and Emotion. *Cereb Cortex*. Jun 1 2019;29(6):2533-2551. doi:10.1093/cercor/bhy123

7. Du J, DiNicola LM, Angeli PA, et al. Organization of the human cerebral cortex estimated within individuals: networks, global topography, and function. *Journal of neurophysiology*. 2024;131(6):1014-1082. doi:10.1152/jn.00308.2023

8. Yeo BT, Krienen FM, Sepulcre J, et al. The organization of the human cerebral cortex estimated by intrinsic functional connectivity. *J Neurophysiol*. Sep 2011;106(3):1125-65. doi:10.1152/jn.00338.2011

9. Holmes AJ, Hollinshead MO, O'Keefe TM, et al. Brain Genomics Superstruct Project initial data release with structural, functional, and behavioral measures. *Sci Data*. 2015;2:150031. doi:10.1038/sdata.2015.31

10. Dale AM, Fischl B, Sereno MI. Cortical surface-based analysis. I. Segmentation and surface reconstruction. *Neuroimage*. Feb 1999;9(2):179-94. doi:10.1006/nimg.1998.0395

11. Jenkinson M, Bannister P, Brady M, Smith S. Improved optimization for the robust and accurate linear registration and motion correction of brain images. *Neuroimage*. Oct 2002;17(2):825-41. doi:10.1016/s1053-8119(02)91132-8

12. Andersson JLR, Skare S, Ashburner J. How to correct susceptibility distortions in spin-echo echo-planar images: application to diffusion tensor imaging. *NeuroImage*. 2003;20(2):870-888. doi:10.1016/s1053-8119(03)00336-7

13. DuPre E, Salo T, Ahmed Z, et al. TE-dependent analysis of multi-echo fMRI with tedana. *Journal of Open Source Software*. 2021;6(66)doi:10.21105/joss.03669

14. Greve DN, Fischl B. Accurate and robust brain image alignment using boundary-based registration. *Neuroimage*. Oct 15 2009;48(1):63-72. doi:10.1016/j.neuroimage.2009.06.060

15. Power JD, Mitra A, Laumann TO, Snyder AZ, Schlaggar BL, Petersen SE. Methods to detect, characterize, and remove motion artifact in resting state fMRI. *Neuroimage*. Jan 1 2014;84:320-41. doi:10.1016/j.neuroimage.2013.08.048

16. Smith SM, Jenkinson M, Woolrich MW, et al. Advances in functional and structural MR image analysis and implementation as FSL. *Neuroimage*. 2004;23 Suppl 1:S208-19. doi:10.1016/j.neuroimage.2004.07.051

medRxiv preprint doi: https://doi.org/10.1101/2025.11.21.25340716; this version posted November 25, 2025. The copyright holder for this preprint (which was not certified by peer review) is the author/funder, who has granted medRxiv a license to display the preprint in perpetuity.

It is made available under a CC-BY 4.0 International license .

17. Gordon EM, Laumann TO, Adeyemo B, Huckins JF, Kelley WM, Petersen SE. Generation and Evaluation of a Cortical Area Parcellation from Resting-State Correlations. *Cereb Cortex*. Jan 2016;26(1):288-303. doi:10.1093/cercor/bhu239
18. Esteban O, Markiewicz CJ, Blair RW, et al. fMRIPrep: a robust preprocessing pipeline for functional MRI. *Nature Methods*. 2018;16(1):111-116. doi:10.1038/s41592-018-0235-4
19. Gorgolewski K, Burns CD, Madison C, et al. Nipype: a flexible, lightweight and extensible neuroimaging data processing framework in python. *Front Neuroinform*. 2011;5:13. doi:10.3389/fninf.2011.00013
20. Lashkari D, Vul E, Kanwisher N, Golland P. Discovering structure in the space of fMRI selectivity profiles. *Neuroimage*. Apr 15 2010;50(3):1085-98. doi:10.1016/j.neuroimage.2009.12.106
21. Woolrich MW, Behrens TE, Smith SM. Constrained linear basis sets for HRF modelling using Variational Bayes. *Neuroimage*. Apr 2004;21(4):1748-61. doi:10.1016/j.neuroimage.2003.12.024
